# Analysis of genetic diversity and phylogenetic relationships among Indonesian native cattle breeds using microsatellite markers: A review

**DOI:** 10.14202/vetworld.2025.1036-1046

**Published:** 2025-04-30

**Authors:** Rini Hastarina, Agung Purnomoadi, Sutopo Sutopo, Dela Ayu Lestari, Fatmawati Mustofa, Putu Novia Gariri, Pupus Galau Prahara, Firda Tasya Kamila, Syaddad Verahry Philco, Maulida Arkaan Muhammad Da’i, Asep Setiaji

**Affiliations:** 1 Department of Animal Science, Faculty of Animal and Agricultural Sciences, Universitas Diponegoro, Tembalang Campus, Semarang, 50275 Central Java, Indonesia; 2 Faculty of Animal Husbandry and Fisheries, Tadulako University, Palu 94148 Central Sulawesi, Indonesia; 3 Bali Cattle National Breeding Center. Jl. Gurita III, Pegok, Sesetan, 80223, Denpasar, Bali, Indonesia

**Keywords:** breed conservation, genetic diversity, inbreeding, Indonesian cattle, microsatellite markers, phylogenetics, sustainable livestock

## Abstract

Indonesia is home to a diverse array of native cattle breeds - such as Aceh, Bali, Madura, and Sumba Ongole - that are uniquely adapted to their regional environments and integral to the country’s agricultural and cultural heritage. This review synthesizes current research on the genetic diversity and phylogenetic relationships of Indonesian native cattle using microsatellite markers, a robust molecular tool for assessing genetic variation. Analysis of multiple studies reveals that geographical isolation, selective breeding, and human-mediated practices have shaped the genetic structure of these populations. Notably, Bali and Madura cattle exhibit distinctive genetic clusters reflecting island isolation and long-standing breeding traditions. Findings also underscore concerns regarding genetic erosion caused by uncontrolled crossbreeding with exotic breeds, which threatens local adaptability and increases the risk of inbreeding. Key genetic parameters - including allele richness, heterozygosity, and inbreeding coefficients - suggest varying degrees of genetic integrity among the breeds, with several populations showing signs of inbreeding depression. Microsatellite data further demonstrate clear phylogenetic separation among breeds, offering valuable insights for breed identification and conservation planning. The primary strength of this review lies in its comprehensive integration of genetic studies across diverse breeds and islands, providing a national-scale perspective. However, limitations include the underrepresentation of certain breeds and reliance on microsatellite data without integration of high-resolution genomic tools. Future research should incorporate advanced molecular techniques e.g., sngle-nucleotide polymorphism arrays and whole-genome sequencing) and longitudinal monitoring to inform targeted conservation strategies. This review advocates for the incorporation of molecular genetic data into national breeding and conservation programs. Strengthening such initiatives is essential for preserving Indonesia’s indigenous cattle as valuable genetic resources for climate-resilient, sustainable livestock production.

## INTRODUCTION

This section provides an overview of the critical importance of preserving genetic diversity within cattle populations, particularly among Indonesia’s native breeds. Genetic diversity is essential for enabling cattle to adapt to climate variability, resist diseases, and maintain long-term sustainability, making it a key asset in modern livestock management. It enhances selective breeding programs by offering a broad array of traits that contribute to improved productivity, health, and environmental resilience. Moreover, conserving genetic diversity helps preserve the unique attributes of native breeds, ensuring flexibility for future breeding initiatives [1–3]. Indigenous Indonesian cattle breeds possess distinct adaptations to local environmental conditions, endemic pathogens, and regional feed resources, giving them a unique advantage in sustainable livestock production [4]. Their genetic diversity not only supports ongoing livestock development but also offers a valuable genetic reservoir tailored to Indonesia’s agricultural systems [5, 6].

Indonesia plays a prominent role in Asia’s genetic livestock resources, housing a wide range of native cattle breeds that are integral to the nation’s cultural heritage and are distinguished by their unique phenotypic and genotypic characteristics [7]. However, despite their significance, these populations face escalating threats from habitat degradation, indiscriminate crossbreeding, and insufficient genetic monitoring programs. Addressing these challenges necessitates a multidisciplinary strategy that combines genetic analysis, breeding technologies, and policy reforms.

Microsatellite markers serve as reliable molecular tools for assessing genetic diversity in livestock species and have been widely employed for this purpose [8–10]. This review examines the current state of genetic diversity and phylogenetic relationships among Indonesia’s native cattle breeds by synthesizing findings from previous studies. These breeds hold considerable genetic and cultural value but are increasingly vulnerable to crossbreeding and environmental pressures. By comparing the genetic structure of Indonesian cattle with local breeds from other countries, this review situates Indonesia’s unique genetic assets within a broader global context. It also underscores the adaptability and resilience of these native breeds, which are vital for promoting sustainable cattle production. Furthermore, several practical strategies are proposed to support the conservation and genetic enhancement of these resources for future breeding and adaptive use.

This review aimed to evaluate the current genetic diversity status of Indonesian native cattle breeds, based on microsatellite marker analyses reported in previous literature. The review highlights the adverse effects of unregulated crossbreeding with exotic breeds, which have resulted in genetic erosion, diminished adaptability, and elevated risks of inbreeding depression in local populations. In response, this review advocates for the integration of molecular genetic data into national breeding programs to strengthen conservation efforts, reduce inbreeding, and enhance the productivity and resilience of Indonesia’s cattle sector. Such an approach not only promotes the long-term viability of native breeds but also reinforces Indonesia’s contribution to global cattle genetic resources. Ultimately, the findings stress the necessity of adopting evidence-based breeding strategies to ensure the genetic resilience, economic performance, and ecological sustainability of Indonesia’s cattle industry in the face of future agricultural challenges.

## CATTLE BREEDING IN INDONESIA AND OVERVIEW OF NATIVE INDONESIAN CATTLE BREEDS

Cattle breeding systems in Indonesia encompass a combination of traditional practices and modern approaches, reflecting efforts to balance productivity-enhancing crossbreeding programs with the preservation of resilient native breeds. Over time, these indigenous cattle have adapted to local environmental pressures - such as high ambient temperatures, endemic diseases, and limited feed resources - demonstrating notable efficiency and adaptability [11]. In many rural areas, cattle serve as essential sources of income and nutrition, playing a pivotal role in the local economy and cultural traditions. Although modernization has driven interest in high-yielding exotic breeds, conserving and utilizing native breeds remains critical for promoting sustainable, locally adapted livestock systems that support food security and ecological resilience [12]. The Indonesian cattle sector faces both opportunities and challenges, especially in addressing the growing demand for beef and enhancing domestic production capacity [13]. Due to the relatively lower meat yield of native breeds, farmers frequently prefer exotic breeds; however, uncontrolled breeding has led to a marked decline in native cattle populations [6].

**Figure 1:**
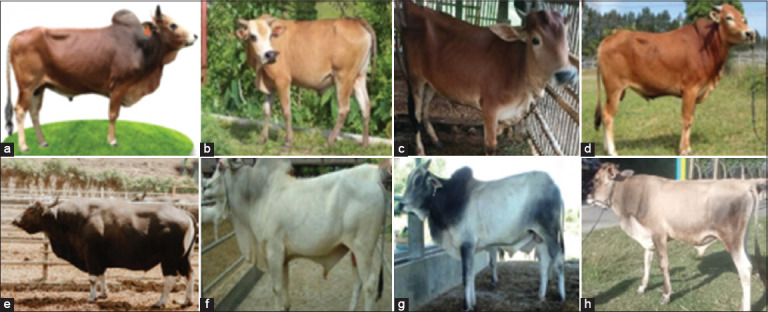
Native cattle breeds in Indonesia (a) Aceh cattle, (b) Pesisir cattle, (c) Pasundan cattle, (d) Madura cattle, (e) Bali cattle, (f) Ongole grade cattle, (g) Sumba Ongole cattle, and (h) Gorontalo cattle.

Indonesia’s native cattle breeds - including Aceh, Pesisir, Pasundan, Madura, Bali, Ongole Grade, Sumba Ongole (SO), and Gorontalo - exhibit distinct genetic profiles shaped by geographic isolation, selective breeding, and adaptation to diverse agro-ecological environments (Figure 1). Each breed presents unique traits aligned with specific regional conditions - for instance, the robust frame and high disease resistance of Bali cattle [14], or the deep-rooted cultural relevance of Madura cattle [15]. These breeds enhance the genetic diversity of Indonesia’s livestock resources and provide traits such as drought tolerance and efficient feed conversion, which are particularly valuable for smallholder farming systems [16, 17]. Although their meat production is generally lower compared to exotic breeds, their resilience to environmental stressors renders them crucial for the sustainability of tropical livestock systems.

### Aceh cattle

Aceh cattle originate from the northern region of Sumatra Island, specifically the Aceh province. This small- to medium-sized breed is well-adapted to tropical climates and low-input farming systems [18]. Characterized by a reddish-brown coat with lighter underbellies, these animals possess a compact and sturdy build, making them ideal for subsistence-level farming [19]. While their meat yield is modest in comparison to exotic breeds, Aceh cattle are heat-tolerant, disease-resistant, and thrive on low-quality forage.

The breed typically has longer calving intervals (18–24 months), a trait that reflects their adaptation to extensive grazing systems and variable feed conditions [20]. Despite a population decline due to crossbreeding, their efficiency under local conditions and high adaptability make them valuable to small-scale farmers. Aceh cattle were formally recognized as an indigenous Indonesian breed in 2011 under Ministry of Agriculture Decree No. 2907/Kpts/OT.140/6/2011 [21].

### Pesisir cattle

Native to West Sumatra, Pesisir cattle represent an important genetic resource within Indonesia [22]. These smaller-bodied cattle are highly valued for their adaptability to both mountainous and coastal regions, particularly in hot climates and under poor forage conditions [23]. Pesisir cattle were officially designated as a native breed by the Ministry of Agriculture in 2011 under Decree No. 2908/Kpts/OT.140/6/2011 [24].

Their physical features include a brick-red coat ranging from yellow to brown, with blonde eyelashes, black markings on the dorsal line, white legs, black tail hair, and small horns. They exhibit a compact build with short limbs, slender legs, and a low body weight - averaging around 160 kg at 4–6 years of age - substantially lower than Bali (310 kg), PO (388 kg), Aceh (302 kg), and Madura cattle (248 kg) [25]. According to the Indonesian National Standard (SNI 7651.6:2015), Pesisir cattle aged 18–24 months typically measure 92 cm in height, 94 cm in body length, and 111 cm in chest girth [26].

### Pasundan cattle

Pasundan cattle, also referred to as “Pakidulan cattle” or “Sapi Rancah” in the Sundanese language, are native to West Java [27]. These cattle are well-suited to diverse altitudes, thriving in both highland and lowland environments. They display a uniform reddish-brown coat in both sexes and are medium-sized, with strong physical endurance, making them suitable for various grazing systems typical of West Java [7].

Pasundan cattle were formally recognized by the Indonesian Ministry of Agriculture in 2014 under Decree No. 1051/Kpts/SR.120/10/2014. In addition to their economic value, they play a significant role in preserving traditional agricultural practices. Their adaptability and suitability to local environmental and economic conditions make them a valuable genetic resource for sustainable livestock development in the region [28].

### Madura cattle

Madura cattle originate from Madura Island, located off the northeastern coast of Java. As described in [29], these cattle exhibit distinctive physical traits: A coat ranging from red to brick-red or brown with white markings on the belly and inner thighs, black tail hair, and no dewlaps. Males have a prominent hump, while females do not. A dorsal stripe and circular markings around the eyes and ears are also commonly observed. Both sexes display consistent coat coloration from birth to adulthood. Their horns are small and curved, with nearly invisible horns in females [30].

Madura cattle are characterized by uniform phenotypic traits, small body size, and short but strong legs. They are essential for rural livelihoods, providing financial, labor, and cultural benefits. In addition to their use as draft animals, they are featured in traditional activities such as *Karapan sapi* (bull racing) and *Sonok* (cattle beauty contests) [31]. Advantages of Madura cattle include high adaptability to tropical climates, efficient growth on poor-quality forage, a high carcass yield, and good meat quality [29].

### Bali cattle

Bali cattle, native to Bali Island, are recognized as the original germplasm of the wild banteng (*Bos sondaicus*), a species indigenous to Southeast Asia [32]. As outlined in the Indonesian National Standard (SNI 7651–4:2023), young males under 18 months typically exhibit a red or blackish coat, while adult males over 18 months are characterized by a solid black coloration. Distinctive phenotypic traits include white “sock-like” markings on the knees, a white half-moon-shaped marking on the underside, and a black tail tip [33], which make this breed readily identifiable.

Bali cattle are highly valued for their hardiness and adaptability to harsh environmental conditions [34]. They exhibit strong disease resistance and can perform well where other breeds might struggle. In addition, Bali cattle demonstrate high fertility rates and maintain satisfactory growth and milk production even on poor-quality forage [35]. These attributes render them a vital asset for smallholder farmers, supporting both meat and milk production and contributing to sustainable agriculture and rural livelihoods in Indonesia [36].

### Ongole-grade cattle

Ongole-grade cattle are regarded as one of Indonesia’s premier beef cattle breeds, particularly well-suited to tropical environments [28]. This breed is the product of crossbreeding between local Indonesian cattle and Indian Ongole cattle, further improved through grading-up programs involving Java and SO cattle since the 1930s [15]. Ongole-grade cattle are distinguished by their large, elongated bodies, short necks, and long limbs, contributing to their substantial size and robust frame [37].

Adult males generally exhibit a white coat with grayish or blackish markings on the head, neck, and hump, along with occasional black patches on the knees. Other identifying features include medium-thick skin, long heads with slightly drooping ears, and short, outward-curving horns. Males possess prominent, upright humps and a large, fleshy dewlap that extends to the lower abdomen near the navel [38].

In addition to their physical robustness, Ongole-grade cattle display exceptional adaptability to suboptimal grazing environments and maintain strong reproductive efficiency under stress [39]. Their resilience and productivity have established them as a core component of Indonesia’s beef production systems, suitable for both intensive and extensive farming contexts [6, 40].

### SO cattle

SO cattle are an indigenous breed developed from Ongole cattle imported to Sumba Island in 1914 [41]. Over time, these animals have adapted to the specific environmental conditions of the island, resulting in a distinct local breed that, while similar to Ongole-grade cattle, exhibits unique traits aligned with the regional ecology [42].

SO cattle play a crucial role in the region’s livestock sector. They are known for their relatively high meat yield and low carcass fat content, making them particularly suitable for fattening programs, especially in feedlot systems. Their ability to accumulate significant weight over extended periods enables them to achieve high slaughter weights, making them ideal candidates for commercial beef production. Their adaptability and efficiency support sustainable livestock practices and contribute to both the local economy and national agricultural development [4].

### Gorontalo cattle

Gorontalo cattle, also known locally as Diiti cattle, are indigenous to the Gorontalo province and share genetic similarities with Ongole-grade cattle. However, the genetic quality of Diiti cattle has reportedly declined due to prolonged inbreeding [43]. Their coat color varies, including white, reddish-brick, and blackish-white.

Distinctive physical features include various horn shapes – V-shaped, U-shaped, straight, and backward-curved - and the universal presence of a dewlap extending from the lower neck to the chest. From a lateral view, both male and female Diiti cattle present either flat or slightly convex facial profiles. The average body weights are approximately 113.75 kg for males and 111.11 kg for females [43].

## GENETIC DIVERSITY AND ITS IMPORTANCE IN BREEDING STRATEGIES

**Figure 2:**
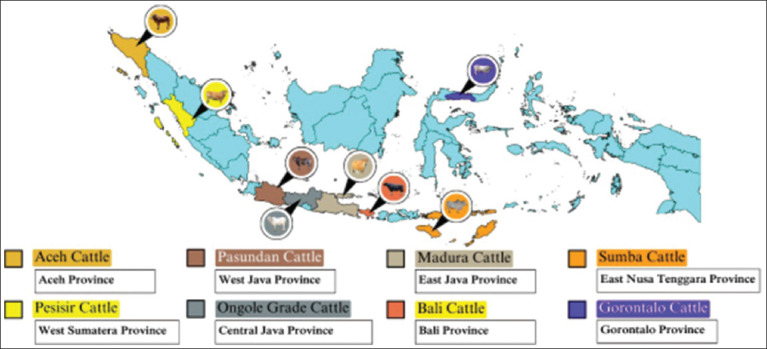
Distribution of native breeds in Indonesia.

Genetic diversity refers to the presence of various alleles and genotypes that result in differences in morphological, physiological, and behavioral traits among individual cattle [44]. It encompasses the total variability within and among subpopulations, varieties, and types within a given cattle species. Preserving genetic diversity is fundamental to maintaining breed viability, enhancing adaptability to environmental challenges, and supporting global food security. In the Indonesian context, safeguarding genetic diversity in local cattle breeds is essential for responding to climate change, shifting consumer preferences, and evolving livestock production systems [4, 45]. Figure 2 illustrates the distribution of native cattle breeds across Indonesia. The conservation of genetic diversity plays a key role in securing long-term food stability and resilience [46]. It serves as a cornerstone for breed adaptability, enabling populations to respond to ecological and economic pressures. However, erosion of genetic diversity, primarily due to inbreeding and genetic bottlenecks - can lead to reduced fertility, diminished disease resistance, and the loss of traits of economic importance.

Maintaining a broad genetic base supports the development of resilient cattle populations aligned with good farming practices (GFP) while ensuring the sustainability and adaptability of livestock resources for future generations. In Indonesia, this genetic variability is increasingly threatened by the dominance of high-yielding exotic breeds and the widespread occurrence of uncontrolled crossbreeding. This trend reflects an overemphasis on enhancing economic traits, often at the cost of conserving native genetic resources [47]. Despite widespread recognition of the importance of conserving animal genetic resources (AnGR), the existence of over 7,500 livestock breeds globally presents logistical challenges for effective conservation, requiring a prioritization framework. Key criteria for prioritizing breeds include productivity, adaptability, population trends, sociocultural importance, geographic distribution, vulnerability to crossbreeding, and the presence of breeder organizations and conservation programs [48]. Political and institutional stability also plays a crucial role in determining the effectiveness of these strategies. Applying such prioritization in Indonesia is essential to preserve local cattle genetic resources while advancing sustainable livestock development.

The overarching goal of conservation programs is to protect as much genetic variation as possible. Priority should be given to native cattle breeds with small populations or those at risk of extinction [49]. Given the declining populations of several Indonesian native cattle breeds - each with unique adaptive traits - it is imperative to implement conservation initiatives. Molecular tools, particularly microsatellite analysis, are essential for characterizing genetic variation and setting conservation priorities. These tools offer valuable insights for designing sustainable breeding programs and safeguarding Indonesia’s rich cattle genetic heritage.

## GENETIC DIVERSITY AND ITS IMPORTANCE IN BREEDING STRATEGIES

Genetic diversity refers to the presence of various alleles and genotypes that result in differences in morphological, physiological, and behavioral traits among individual cattle [44]. It encompasses the total variability within and among subpopulations, varieties, and types within a given cattle species. Preserving genetic diversity is fundamental to maintaining breed viability, enhancing adaptability to environmental challenges, and supporting global food security. In the Indonesian context, safeguarding genetic diversity in local cattle breeds is essential for responding to climate change, shifting consumer preferences, and evolving livestock production systems [4, 45]. Figure 2 illustrates the distribution of native cattle breeds across Indonesia. The conservation of genetic diversity plays a key role in securing long-term food stability and resilience [46]. It serves as a cornerstone for breed adaptability, enabling populations to respond to ecological and economic pressures. However, erosion of genetic diversity - primarily due to inbreeding and genetic bottlenecks - can lead to reduced fertility, diminished disease resistance, and the loss of traits of economic importance.

Maintaining a broad genetic base supports the development of resilient cattle populations, aligned with GFP, while ensuring the sustainability and adaptability of livestock resources for future generations. In Indonesia, this genetic variability is increasingly threatened by the dominance of high-yielding exotic breeds and the widespread occurrence of uncontrolled crossbreeding. This trend reflects an overemphasis on enhancing economic traits, often at the cost of conserving native genetic resources [47]. Despite widespread recognition of the importance of conserving AnGR, the existence of over 7,500 livestock breeds globally presents logistical challenges for effective conservation, requiring a prioritization framework. Key criteria for prioritizing breeds include productivity, adaptability, population trends, sociocultural importance, geographic distribution, vulnerability to crossbreeding, and the presence of breeder organizations and conservation programs [48]. Political and institutional stability also plays a crucial role in determining the effectiveness of these strategies. Applying such prioritization in Indonesia is essential to preserve local cattle genetic resources while advancing sustainable livestock development.

The overarching goal of conservation programs is to protect as much genetic variation as possible. Priority should be given to native cattle breeds with small populations or those at risk of extinction [49]. Given the declining populations of several Indonesian native cattle breeds - each with unique adaptive traits - it is imperative to implement conservation initiatives. Molecular tools, particularly microsatellite analysis, are essential for characterizing genetic variation and setting conservation priorities. These tools offer valuable insights for designing sustainable breeding programs and safeguarding Indonesia’s rich cattle genetic heritage.

## MOLECULAR TECHNIQUES TO DETECT GENETIC DIVERSITY

The success of both breeding and conservation strategies relies heavily on understanding the genetic diversity among cattle breeds. Hailu and Getu [50] utilized phenotypic assessments and chromosomal karyotyping to examine genetic variation. However, the advent of polymerase chain reaction revolutionized the field, enabling the development of molecular techniques such as random amplified polymorphic DNA, restriction fragment length polymorphism, amplified fragment length polymorphism, and microsatellite DNA analysis for investigating genetic diversity in livestock populations [51–53].

Among these, microsatellite markers are the most extensively applied due to their high polymorphism, codominant inheritance, and widespread distribution across coding and noncoding regions of the eukaryotic genome [54, 55]. These short tandem repeats, also known as simple sequence repeats, vary in repeat number across individuals and breeds, making them excellent markers for diversity studies. Microsatellites are highly effective for distinguishing genetic differences among breeds and populations, due to their high mutation rates and reproducibility. The use of molecular markers to characterize AnGR is recognized as a strategic priority globally.

Microsatellites have been widely employed in studies assessing the genetic diversity of local cattle breeds across Asia, including China [56, 57], Japan [58, 59], Korea [60, 61], India [62–63], and Turkey [64, 65]. In Indonesia, microsatellite-based approaches have proven instrumental in evaluating the genetic variability and uniqueness of indigenous cattle populations [4, 66].

## GENETIC DIVERSITY AMONG INDONESIAN NATIVE CATTLE

The first genetic studies employing microsatellite markers on Indonesian native cattle - including Aceh, Pesisir, Ongole, Madura, and Bali breeds - were conducted by [11]. Although allele distributions are not shown here, the results revealed that Aceh, Pesisir, and Filial Ongole cattle are genetically close to Indian zebu breeds, yet intermediate between zebu and Bali cattle. Interestingly, Madura and Galekan cattle, which possess banteng mitochondrial DNA, are genetically more similar to Bali cattle than to zebu breeds. Bali cattle populations themselves appear genetically homogeneous. When genetic distances are analyzed in relation to captive banteng, they show proximity to Bali cattle, likely due to inbreeding effects (data not shown) [11].

According to Sutarno *et al*. [41], five microsatellite markers were employed to analyze the genetic structure of four native breeds - Ongole Grade, Madura, Bali, and Aceh. The study reported a mean observed number of alleles (Na) of 4.20, a mean effective number of alleles (Ne) of 2.38, and a mean polymorphism information content of 0.55. In another study, Agung *et al*. [66] used 12 microsatellite markers and identified a total of 862 alleles. Notably, allele 205 (INRA23) was unique to SO cattle, while allele 219 was specific to Ongole Grade cattle. The mean observed number of alleles ranged from 4.759 in banteng to 9.833 in Ongole Grade. Expected heterozygosity values varied from 0.555 (banteng) to 0.788 (Ongole Grade). The authors highlighted that local Indonesian cattle exhibited a higher mean number of alleles than indigenous cattle breeds elsewhere. Heterozygosity was identified as the most reliable parameter for evaluating genetic variability, although estimates based on average heterozygosity across multiple loci offer a more comprehensive assessment of population-level genetic diversity.

Based on the data in Table 1 [11, 41, 66–69], several cattle populations exhibit negative and positive Fis values, indicating conditions of outbreeding and inbreeding, respectively. Populations with Fis values <0 are classified as experiencing outbreeding, which suggests increased genetic diversity and reduced relatedness among individuals. In contrast, populations with Fis values >0 are indicative of inbreeding, reflecting higher genetic similarity and potential risks associated with inbreeding depression. Notable examples of inbred populations include Pesisir, Pasundan, Madura, Ongole Grade, and SO cattle, as reported by Agung *et al*. [70] and Agung *et al*. [66]. Bali cattle have also been identified as being under inbreeding pressure, supported by studies from Septian *et al*. [68], Dako *et al*. [69] and Agung *et al*. [66]. Similarly, Gorontalo cattle have been categorized as inbred based on findings from [69].

**Table 1:** Genetic diversity parameters of native Indonesian cattle.

Populations	Na	Ne	Ho	He	PIC	Fis*	Reference
Aceh	2.60	2.032	0.85	0.5725	-	−0.4847	[41]
Pesisir	6.36	3.590	0.566	0.704	0.589	0.19602	[66]
Pasundan	7.75	4.705	0.501	0.74	-	0.32297	[66]
Madura	8.42	5.283	0.587	0.796	-	0.26256	[66]
	3.80	2.776	0.930	0.640	-	−0.4531	[41]
Bali	8.00	4.160	0.418	0.604	0.579	0.30795	[68]
	-	-	0.610	0.580	-	−0.0517	[11]
	4.67	3.563	0.611	0.671	0.627	0.08942	[69]
	5.46	3.361	0.632	0.648	0.542	0.02469	[66]
	2.70	2.002	0.635	0.441	-	−0.4399	[41]
Ongole Grade	9.83	5.067	0.651	0.788	0.749	0.17386	[66]
	3.00	2.657	0.667	0.593	0.518	−0.1248	[69]
	3.00	2.248	0.660	0.444	-	−0.4865	[41]
Sumba Ongole	7.08	3.076	0.536	0.615	0.572	0.12846	[66]
	8.17	3.355	0.553	0.662	0.619	0.16465	[66]
Gorontalo	7.00	3.759	0.524	0.716	0.674	0.26816	[69]

* Fis=(He-Ho)/He. Bertorelle [67]. PIC=Polymorphism information content

These results underscore the critical importance of ongoing genetic monitoring and management to mitigate inbreeding-related risks within these populations. The majority of cattle populations analyzed by Agung *et al*. [66] were found to exhibit characteristics consistent with inbreeding, as evidenced by a higher observed heterozygosity relative to the expected heterozygosity. Several factors may contribute to this discrepancy, including the presence of null alleles, assortative (non-random) mating, population subdivision (Wahlund effect), heterozygote disadvantage, and inbreeding itself, or a combination of these influences [71]. Each of these factors can significantly impact genetic variation and population structure, potentially resulting in deviations from Hardy-Weinberg equilibrium.

## PHYLOGENETIC RELATIONSHIPS AMONG INDONESIAN NATIVE CATTLE

The phylogenetic relationships among Indonesia’s native cattle breeds have been extensively investigated using microsatellite markers, which serve as a powerful tool for assessing genetic diversity and evolutionary patterns. Numerous studies have employed these markers to elucidate the genetic structure and relatedness among various Indonesian breeds, such as Bali, Madura, and Ongole Grade, which are recognized for their adaptability to a wide range of environmental conditions [72]. Microsatellite markers, being highly polymorphic and widely dispersed across the genome, offer detailed insights into intra- and inter-breed genetic variation, thereby enhancing our understanding of breed evolution and regional differentiation [73].

A major conclusion from these studies is the clear genetic differentiation observed among native cattle breeds, often shaped by geographic isolation and region-specific breeding practices. For instance, research on Bali cattle – endemic to Bali Island – has demonstrated the presence of distinct genetic clusters that differentiate them from other breeds across Indonesia [68]. Similarly, Madura cattle, native to Madura Island, exhibit unique genetic signatures that reflect their historical separation from other regional cattle populations [41, 74]. These patterns underscore the influence of both natural and anthropogenic factors, including island isolation and targeted breeding, on the genetic architecture of Indonesian native cattle.

Moreover, comparative microsatellite analyses have emphasized the potential benefits of incorporating phylogenetic data into national breeding and conservation programs [41]. Understanding genetic distances and degrees of relatedness among native cattle populations enables the development of informed strategies to preserve valuable genetic traits and enhance breed performance. Such insights can also guide the identification of cattle breeds with specific adaptations to environmental stressors - such as disease resistance and heat tolerance - thus promoting sustainable utilization of Indonesia’s genetic livestock resources [75].

## CONCLUSION

This review compiled genetic data from multiple studies involving microsatellite analyses of Indonesian native cattle breeds, including Bali, Madura, Aceh, Pesisir, Ongole Grade, SO, and Gorontalo. The findings demonstrated moderate to high levels of genetic diversity across most breeds, with observed heterozygosity generally exceeding expected values. Nevertheless, several populations exhibited positive inbreeding coefficients (Fis >0), notably Pesisir, Madura, Ongole Grade, SO, Bali, and Gorontalo cattle, indicating a degree of inbreeding that may compromise long-term genetic health. Phylogenetic assessments revealed distinct clustering patterns, with Bali cattle closely associated with *Bos javanicus* (banteng) ancestry, while Madura and Galekan cattle presented intermediate genetic profiles between zebu and Bali types. These patterns reflect the influence of historical isolation, selective breeding practices, and local adaptation.

Overall, the study confirms that Indonesia’s native cattle breeds harbor significant genetic variability and distinct evolutionary lineages. However, ongoing genetic erosion due to uncontrolled crossbreeding and shrinking population sizes poses a critical threat to their conservation. The application of microsatellite markers has proven valuable in characterizing genetic diversity and informing breeding and conservation strategies.

A notable strength of this review is its comprehensive integration of genetic data across multiple native breeds and its emphasis on phylogenetic relationships, providing insights into evolutionary history and conservation priorities. The analysis also benefits from contextualizing Indonesian data within broader regional and global frameworks. Nonetheless, limitations exist, due to inconsistencies in the choice of microsatellite markers across studies, limited representation of some native breeds, and the absence of high-resolution genomic data. These factors constrain the resolution and comparability of genetic diversity assessments.

Future research should incorporate genome-wide approaches such as SNP arrays and next-generation sequencing to address these gaps to refine genetic characterizations. Longitudinal genetic monitoring will also be essential to assess the impact of breeding programs and conservation interventions over time. Furthermore, integrating molecular data with phenotypic and ecological information will enhance selective breeding strategies to improve resilience, productivity, and adaptability. Institutional and policy support is imperative to develop and implement evidence-based conservation frameworks that preserve Indonesia’s rich and unique cattle genetic resources for sustainable livestock development.

## AUTHORS’ CONTRIBUTIONS

RH and AS: Carried out the conception of the review article. RH, FM, PGP, PNG, FTK, MAMD, and SVP: Drafted the manuscript. AS, DAL, AP, and SS: Supervised the review. All authors have read and approved the final version of the manuscript.

## References

[1] Hoffmann I (2011). Livestock biodiversity and sustainability. Livest. Sci.

[2] Naskar S, Gowane G.R, Chopra A, Sejian V, Gaughan J, Baumgard L, Prasad C (2015). Strategies to improve livestock genetic resources to counter climate change impact. Climate Change Impact on Livestock: Adaptation and Mitigation.

[3] Eusebi P.G, Martinez A, Cortes O (2020). Genomic tools for effective conservation of livestock breed diversity. Diversity.

[4] Widyas N, Widi T.S.M, Prastowo S, Sumantri I, Hayes B.J, Burrow H.M (2022). Promoting sustainable utilization and genetic improvement of Indonesian local beef cattle breeds: A review. Agriculture.

[5] Sutarno S, Setyawan A.D (2016). The diversity of local cattle in Indonesia and the efforts to develop superior indigenous cattle breeds. Biodiversitas.

[6] Widi T.S.M, Udo H, Oldenbroek K, Budisatria I.G.S, Baliarti E, der Zijpp A.V (2021). Designing genetic impact assessment for crossbreeding with exotic beef breeds in mixed farming systems. Outlook Agric.

[7] Suryaka Y, Sutopo S, Setiaji A (2024). The phenotypic characteristics and morphology of Jabres and Pasundan cattle differences. Biodiversitas.

[8] Hoshino A.A, Bravo J.P, Nobile P.M, Morelli K.A (2012). Microsatellites as tools for genetic diversity analysis. Genetic Diversity in Microorganisms.

[9] Sheriff O, Alemayehu K (2018). Genetic diversity studies using microsatellite markers and their contribution in supporting sustainable sheep breeding programs: A review. Cogent Food Agric.

[10] Olschewsky A, Hinrichs D (2021). An overview of the use of genotyping techniques for assessing genetic diversity in local farm animal breeds. Animals (Basel).

[11] Mohamad K, Olsson M, van Tol H.T, Mikko S, Vlamings B.H, Andersson G, Rodríguez-Martínez H, Purwantara B, Paling R.W, Colenbrander B, Lenstra J.A (2009). On the origin of Indonesian cattle. PLoS One.

[12] Felius M, Theunissen B, Lenstra J.A (2015). Conservation of cattle genetic resources: The role of breeds. J. Agric. Sci.

[13] Agus A, Widi T.S.M (2018). Current situation and future prospects for beef cattle production in Indonesia-A review. Asian-Australas. J. Anim. Sci.

[14] Barboni P, Thompson I, Brownlie J, Hartaningsih N, Collins M.E (2001). Evidence for the presence of two bovine lentiviruses in the cattle population of Bali. Vet. Microbiol.

[15] Widi T.S.M, Udo H.M.J, Oldenbroek K, Budisatria I.G.S, Baliarti E, Van Der Zijpp A.J (2013). Unique cultural values of Madura cattle: Is cross-breeding a threat?. Anim. Genet. Resour.

[16] Nyamushamba G.B, Mapiye C, Tada O, Halimani T.E, Muchenje V (2017). Conservation of indigenous cattle genetic resources in Southern Africa's smallholder areas: Turning threats into opportunities-A review. Asian-Australas. J. Anim. Sci.

[17] Vanvanhossou S.F.U, Dossa L.H, König S (2021). Sustainable management of animal genetic resources to improve low-input livestock production: Insights into local Beninese cattle populations *Sustainability*.

[18] Saputra R, Rusdi M., Samadi S (2021). Analysis of land suitability for Aceh cattle based on environmental physical characteristics (case study in Aceh Besar district). IOP Conf. Ser. Earth Environ. Sci.

[19] Abdullah M.A.N, Noor R.R, Martojo H, Solihin D.D, Handiwirawan E (2007). The phenotypic variability of Aceh Cattle in Nanggroe Aceh Darussalam. J. Indones. Trop. Anim. Agric.

[20] Budisatria I.G.S, Baliarti E, Widi T.S.M, Ibrahim A, Atmoko B.A (2019). Reproductive management and performances of Aceh cows, local Indonesian cattle kept by farmers in a traditional system. Am. Eurasian J. Sustain. Agric.

[21] Ministry of Agriculture of the Republic of Indonesia (2014). Ministerial Decree No. 427/Kpts/SR.120/3/2014 Tentang Penetapan Rumpun Sapi Sumba Ongole.

[22] Afriani T, Udin Z, Hellyward J, Purwati E, Rastosari A, Wahyudi D (2022). Separation of bull spermatozoa bearing X-and Y-chromosomes by using albumin gradient and swim-up technique in pesisir cattle. J. Anim. Health. Prod.

[23] Pazla R, Adrizal S.R (2021). Intake, nutrient digestibility and production performance of Pesisir cattle fed *Tithonia diversifolia* and *Calliandra calothyrsus*-based rations with different protein and energy ratios. Adv. Anim. Vet. Sci.

[24] Budhiyadnya I.G.E, Udin Z, Purwati E, Yellita Y (2021). The effect of age, body height, weight, testosterone hormone concentration and semen quality on the libido level of pesisir cattle. J. Anim. Health Prod.

[25] Putri A.E, Farajallah A, Perwitasari D (2019). The origin of Pesisir cattle based on D-loop mitochondrial DNA. Biodiversitas.

[26] Badan Standardisasi Nasional (2015). SNI 7651.6.2015. Bibit Sapi Potong Bagian 6: Pesisir. 2015, ICS 65.020.30 BSN [Indonesia].

[27] Said S, Putra W.P.B, Anwar S, Agung P.P, Yuhani H (2017). Phenotypic, morphometric characterization and population structure of Pasundan cattle at West Java, Indonesia. Biodiversitas.

[28] Warman A.T, Panjono P, Fadhilah G.T, Atmoko B.A, Bintara S, Widi T.S.M, Jannah Z.N (2024). The difference between Bali cattle and Limousin-Bali (Limbal) crossed cattle concerning their qualitative characteristics in Lombok Tengah District, Indonesia. Nusantara Biosci.

[29] Nurgiartiningsih V.M.A, Budiarto A, Kusmartono K, Suyadi S (2016). Evaluation of performance in female Madura cattle in Madura Island, Indonesia. Anim. Prod.

[30] Maylinda S, Nugroho H, Busono W (2017). Phenotypic characteristics of local cattle in Madura Island. AIP Conf. Proc.

[31] Prihandini P.W, Maharani D, Sumadi S (2020). Body weight, body measurements and slaughter characteristics of Madura cattle raised in Pamekasan District, East Java Province, Indonesia. Biodiversitas.

[32] Abadi M, Nafiu L.O, Saili T, Yunus L, Iswandi R.M, Rianda L, Pagala M.A, Alwi L.O, Sani L.O.A (2024). Mapping Bali cattle breeding centers (Case study: South Konawe, Indonesia). IOP Conf. Ser. Earth Environ. Sci.

[33] Purwantara B, Noor R.R, Andersson G, Rodriguez-Martinez H (2012). Banteng and Bali cattle in Indonesia: Status and forecasts. Reprod. Domest Anim.

[34] Sikone H.Y, Bouk G, Bere E.K, Kamlasi Y, Nugraha E.Y (2024). Comparative study of production performance and income of bali cattle farmers at different altitudes and maintenance typologies. Adv. Anim. Vet. Sci.

[35] Marawali H.H, Ratnawaty S (2015). The effect of Timor Island legumes on body weight gain of post weaning Bali cattle. J. Agric. Sci. Technol.

[36] Mohamad K, Olsson M, Andersson G, Purwantara B, Van Tol H.T.A, Rodriguez-Martinez H, Colenbrander B, Lenstra J.A (2012). The origin of Indonesian cattle and conservation genetics of the Bali cattle breed. Reprod. Domest. Anim.

[37] Ngadiyono N, Panjono P, Budhi S.P.S, Susanti A.E (2017). Characteristics of Ongole Grade Cows in the Kebumen Regency, Central Java Province. Proceedings of the 7^th^International Seminar on Tropical Animal Production.

[38] Williamson G, Payne W.J.A (1978). An Introduction to Animal Husbandry in the Tropics.

[39] Widodo W, Kamardiani D.R, Utami B.N (2022). Behavioral response of breeder toward development program of Ongole crossbred cattle in Yogyakarta Special Region, Indonesia. Open Agric.

[40] Bramastya T.A, Sukaryo S, Dhiaurridho M.I, Riyanto J, Volkandari S.D, Sudrajad P, Cahyadi M (2022). Characteristics of body weight and measurement of Peranakan Ongole and Brahman cattle in the tropics. IOP Conf. Ser. Earth Environ. Sci.

[41] Sutarno S, Setyawan A.D, Lymbery A.J (2015). Genetic diversity of five Indonesian native cattle breeds at microsatellite loci. Asian J. Anim. Sci.

[42] Sudrajad P, Hartati H, Soewandi B.D.P, Anwar S, Hapsari A.A.R, Widi T.S.M, Bintara S, Maharani D (2023). Population diversity, admixture, and demographic trend of the Sumba Ongole cattle based on genomic data. Anim. Biosci.

[43] Dako S, Laya N, Fathan S, Gubali S, Datau F, Syahruddin S, Pateda S.Y (2024). Phenotype characteristics of Diiti cattle in the coastal region of Tomini bay-Gorontalo, Indonesia. Yuzuncu Yıl Univ. J. Agric. Sci.

[44] Toro M.A, Caballero A (2005). Characterization and conservation of genetic diversity in subdivided populations. Philos. Trans. R. Soc. Lond. B Biol. Sci.

[45] Seré C, van der Zijpp A, Persley G, Rege E (2008). Dynamics of livestock production systems, drivers of change and prospects for animal genetic resources. Anim. Genet. Resour. Inf.

[46] Dumont B, Puillet L, Martin G, Savietto D, Aubin J, Ingrand S, Thomas M (2020). Incorporating diversity into animal production systems can increase their performance and strengthen their resilience. Front. Sustain. Food Syst.

[47] Darmawan H, Chang H. L, Wu H.H (2023). A community-based breeding program as a genetic resource management strategy of Indonesian Ongole cattle. Sustainability.

[48] Mathew E, Mathew L, Sukumaran S.T, Keerthi T.R (2023). Conservation of landraces and indigenous breeds: An investment for the future. Conservation and Sustainable Utilization of Bioresources. Sustainable Development and Biodiversity.

[49] Zhang M, Peng W.F, Hu X.J, Zhao Y.X, Lv F.H, Yang J (2018). Global genomic diversity and conservation priorities for domestic animals are associated with the economies of their regions of origin. Sci. Rep.

[50] Hailu A, Getu A (2015). Breed characterization: Tools and their applications. Open Access Library J.

[51] Grover A, Sharma P.C (2016). Development and use of molecular markers: Past and present. Crit. Rev. Biotechnol.

[52] Levin R.E, Ekezie F.G.C, Sun D.W (2018). DNA-based technique: Polymerase Chain Reaction (PCR). Modern Techniques for Food Authentication.

[53] Babu K.N, Sheeja T.E, Minoo D, Rajesh M.K, Samsudeen K, Suraby E.J, Kumar I.P.V (2021). Random amplified polymorphic DNA (RAPD) and derived techniques. Methods Mol Biol.

[54] Vieira M.L.C, Santini L, Diniz A.L, Munhoz C.D.F (2016). Microsatellite markers: What they mean and why they are so useful. Genet. Mol. Biol.

[55] Bagshaw A.T (2017). Functional mechanisms of microsatellite DNA in eukaryotic genomes. Genome Biol. Evol.

[56] Mao Y, Chang H, Yang Z, Zhang L, Xu M, Chang G, Sun W, Song G, Ji D (2008). The analysis of genetic diversity and differentiation of six Chinese cattle populations using microsatellite markers. J. Genet. Genomics.

[57] Sun W, Chen H, Lei C, Lei X, Zhang Y (2008). Genetic variation in eight Chinese cattle breeds based on the analysis of microsatellite markers. Genet. Sel. Evol.

[58] Nagamine Y, Nirasawa K, Takahashi H, Sasaki O, Ishii K, Minezawa M, Oda S, Visscher P.M, Furukawa T (2008). Estimation of the time of divergence between Japanese Mishima Island cattle and other cattle populations using microsatellite DNA markers. J. Hered.

[59] Nishimaki T, Ibi T, Tanabe Y, Miyazaki Y, Kobayashi N, Matsuhashi T, Akiyama T, Yoshida E, Imai K, Matsui M, Uemura K, Watanabe N, Fujita T, Saito Y, Komatsu T, Yamada T, Mannen H, Sasazaki S, Kunieda T (2013). The assessment of genetic diversity within and among the eight subpopulations of Japanese Black cattle using 52 microsatellite markers. Anim. Sci. J.

[60] Shi Z, Lee J.H, Lee Y.S, Oh D.Y, Yeo J.S (2010). Analysis of genetic diversity and distances in Asian cattle breeds using microsatellite markers. J. Korean Data Inf. Sci. Soc.

[61] Suh S, Kim Y.S, Cho C.Y, Byun M.J, Choi S.B, Ko Y.G, Lee C.W, Jung K.S, Bae K.H, Kim J.H (2014). Assessment of genetic diversity, relationships and structure among Korean native cattle breeds using microsatellite markers. Asian-Australas J. Anim. Sci.

[62] Sodhi M, Mukesh M, Mishra B.P, Ahlawat S.P.S, Prakash B, Sobti R.C (2011). Microsatellite analysis of genetic population structure of Zebu cattle (*Bos indicus*) breeds from North-Western region of India. Anim. Biotechnol.

[63] Sharma R, Kishore A, Mukesh M, Ahlawat S, Maitra A, Pandey A.K, Tantia M.S (2015). Genetic diversity and relationship of Indian cattle inferred from microsatellite and mitochondrial DNA markers. BMC Genet.

[64] Demir E, Balcioglu M.S (2019). Genetic diversity and population structure of four cattle breeds raised in Turkey using microsatellite markers. Czech J. Anim. Sci.

[65] Özşensoy Y.U.S.U.F, Kurar E, Doğan M, Bulut Z, Ni-Zamlioğlu M, Isik A, Camlidag A, Altunok V (2019). Phylogenetic relationships of native Turkish cattle breeds using microsatellite markers. Turk. J. Vet. Anim. Sci.

[66] Agung P.P, Saputra F, Zein M.S.A, Wulandari A.S, Putra W.P.B, Said S, Jakaria J (2019). Genetic diversity of Indonesian cattle breeds based on microsatellite markers. Asian-Australas. J. Anim. Sci.

[67] Bertorelle G (2009). Population Genetics for Animal Conservation.

[68] Septian W.A, Jakaria J, Sumantri C (2015). Genetic diversity of Bali cattle based on microsatellite marker in Indonesian breeding centre. Med. Peternakan.

[69] Dako S, Laya N.K, Gubali S.I, Ardiantoro A, Nurgiartiningsih V.M.A, Ciptadi G, Wulandari D, Suyadi S (2023). Genetic diversity of Gorontalo local cattle based on microsatellite DNA. Adv. Anim. Vet. Sci.

[70] Agung P.P, Anwar S, Wulandari A.S, Sudiro A, Said S, Tappa B (2015). The potency of Sumba Ongole (SO) cattle: A study of genetic characterization and carcass productivity. J. Indones. Trop. Anim. Agric.

[71] Cervini M, Henrique S.F, Mortari N, Matheucci E (2006). Genetic variability of 10 microsatellite markers in the characterization of Brazilian Nellore cattle (*Bos indicus*). Genet. Mol. Biol.

[72] Putra W.P.B, Noor R.R, Sumantri C, Margawati E.T (2024). Genetic characterization in four Indonesian cattle breeds inferred from illumina parentage SNP markers. Iraqi J. Agric. Sci.

[73] Sudrajad P, Subiharta S, Adinata Y, Lathifah A, Lee J.H, Lenstra J.A, Lee S.H (2020). An insight into the evolutionary history of Indonesian cattle assessed by whole genome data analysis. PLoS One.

[74] Hartatik T, Volkandari S.D, Sumadi S, Widodo W (2013). The application of polymerase chain reaction-restriction fragment length polymorphisms (PCR-RFLP) to determine genetic diversity of Madura cattle in Sapudi Island. Indones J. Biotechnol.

[75] Yaro M, Munyard K.A, Stear M.J, Groth D.M (2017). Molecular identification of livestock breeds: A tool for modern conservation biology. Biol. Rev Camb Philos Soc.

